# Benefits, Satisfaction and Limitations Derived from the Performance of Intergenerational Virtual Activities: Data from a General Population Spanish Survey

**DOI:** 10.3390/ijerph19010401

**Published:** 2021-12-30

**Authors:** Alejandro Canedo-García, Jesús-Nicasio García-Sánchez, Deilis-Ivonne Pacheco-Sanz

**Affiliations:** 1 Department of Psychology, Sociology and Philosophy, Universidad de León, 24071 Leon, Spain; acang@unileon.es; 2 Department of Psychology, Universidad de Valladolid, 47011 Valladolid, Spain; deilisivonne.pacheco@uva.es

**Keywords:** intergenerational interactions, benefits, satisfaction, limitations, ICTs, virtual tools

## Abstract

The growing social gap between people of different generations has led to a greater interest in the study of intergenerational interactions. Digital technologies have become necessary for people of all ages to perform daily activities, increasingly including older people. The use of information and communication technologies (ICTs) and virtual tools can provide older people with excellent opportunities to connect with other generations, improving their quality of life and well-being. The aim of this study was to examine the benefits, satisfaction, and limitations of intergenerational interactions generated by the use of virtual tools. The participants are subjects of any age and different social groups residing in Spain and have completed an online survey. The analysis of sociodemographic data of the respondents showed that there is a significant correlation between the use of social networks and all the variables analyzed, except for their level of autonomy. Most participants who participated in intergenerational virtual activities reported the benefits of their social participation, relationships, mood, mental health, and academic education. Moreover, most participants were quite or very satisfied with the person with whom they used the virtual tools, especially if the person was a friend, their partner, sibling, another relative, or colleague. Except for grandparents, people who participated in intergenerational virtual activities and who had no limitations or disabilities were more frequently reported by the participants. In conclusion, intergenerational interactions through the use of virtual tools can contribute to improving the social inclusion and relationships of all people involved.

## 1. Introduction

In recent decades, there has been a remarkable increase in the number of older people (persons aged over 65 years) worldwide [1]. Currently, adults aged 65 and over represent 8.5% of the world’s population, and it is estimated that this number will double, and older adults will comprise 16.7% of the population by 2050, resulting in 1.6 billion older adults worldwide [2]. This fact has led to a marked interest from different fields of knowledge in active aging and what this concept entails, including the promotion of well-being, quality of life, and active participation in society [3].

Therefore, with population aging, demographic trends, such as the geographic dispersion of families, and changes in family structures, social isolation will continue to be a problem in the future [4]. This has been shown most clearly during the COVID-19 pandemic, where having to stay at home reduced face-to-face interactions and participation in social activities. Recent studies have described the possible implications of social restrictions on people’s mental health and well-being [5,6].

Precisely in this context, the use of information and communication technologies (ICT) to search for information, social contact, and leisure activities are increasing in adults and the elderly, which is helping to maintain ties relatives, becoming very important as the geographical separation between family members increases [7]. In this sense, the use of technology by older people is increasing [8,9,10,11,12,13].

Although the increasing digitization of society has been identified as a risk factor that could reduce social inclusion and weaken social ties, due to its potential reduction in face-to-face contact [14], many researchers believe that the new technologies can be useful tools to exchange information, collaborate and favor social connections and, therefore, improve the quality of life and well-being of all population groups [13,15]. Thus, digital technologies, which have become an integral part of daily activities, can offer a mechanism to improve intergenerational relationships and promote the social inclusion of older people [11,16,17].

The use of ICTs is universal and is part of the social life of adolescents, young people, and adults [18] and, increasingly, of older people, in which it has been shown that the daily use of web 2.0 tools improves their quality of life [19]. In this way, social networks and instant messaging apps could become useful virtual tools to favor the development and implementation of intergenerational programs, as they have been shown effective during the course of the COVID-19 pandemic [20,21].

A systematic review was previously carried out in order to identify the relevant elements that ensure the effectiveness of intergenerational interventions, both face-to-face and virtual [22]. The results showed the need to increase the number of virtual interventions that can improve the effectiveness of intergenerational activities. Therefore, we hypothesize that a better understanding of the social interaction through ICTs between people of different generations will be of great interest when it comes to exploring their personal and motivational factors and that it will help to advance toward a society in which all individuals have a place regardless of their age.

The aim of this study was to assess the benefits, satisfaction, and limitations of the intergenerational relationships derived from the performance of virtual activities, using a general population survey in subjects of all ages living in Spain.

## 2. Materials and Methods

### 2.1. Sample

Participants in this study were subjects living in Spain of any age and from different social groups (birthplace, education, marital status, employment situation, income level, etc.) who completed an online questionnaire. A total of 2013 individuals (608 men and 1405 women, 30.2% and 69.8%, respectively) completed the survey and were included in the final study. The mean age of participants was 33.96 years (SD = 16.01) and ranged from 10 to 85 years old. The participants were also grouped into 3 groups of age: <22, 22–39, and ≥40 years old.

### 2.2. Instrument

This study used the previously validated Acción Conjunta Intergeneracional (ACIG) instrument [23]. It consists of an online questionnaire that, through 6 scales and 14 sub-scales, analyzes the information provided by people of all ages in relation to the intergenerational virtual activities they perform with family, friends, acquaintances, or professionals, and a series of psychosocial variables to study the benefits, the satisfaction, and the limitations derived from the performance of programs or activities between generations. Sociodemographic data of participants (age, sex, place of origin, marital status, educational level, autonomy level, living arrangements, employment situation, income level) were also collected by the questionnaire.

Virtual activities were included in four groups: (1) activities with virtual communication tools (Skype, WhatsApp, E-mail, etc.), (2) activities with virtual educational tools (Moodle, WebQuest, Blogs, Wikis, Microsoft Word, Excel, PowerPoint, YouTube, etc.), (3) activities with web browsers (Google, Mozilla, Internet Explorer, Google Drive, Dropbox, OneDrive, Google Earth, Google Maps, etc.), and (4) activities with social networks (Facebook, Twitter, Instagram, Google+, etc.). For each group of activities, participants were asked the questions shown in Table 1. Responses were recategorized when required for the statistical analysis. The part of the instrument related to virtual activities, at the online survey, can be found as Supplementary Material.

The validation of the instruments was carried out in two phases. First, exploratory factor analyzes were performed with half of the EFA sample, with the SPSS v26 software verifying the validity of the construct. From the standard matrices obtained, the MacDonald compound/omegas reliabilities were calculated in Excel, being greater than 0.90; the average variances extracted AVE (or convergent validity), being higher than 0.50; and the discriminant validity (square of the AVE) being greater than the intercorrelations between the factors. Second, with the other half of the sample, the CFA confirmatory factor analysis, using the AMOS v26 software; from the EFA pattern matrices, checking the adequacy of the measurement model (construct validity), with RMSEA coefficients less than 0.08; CFI and TLI above 0.90. Again, using the Gazkin plugins (WikStat Gazkination), compound reliability, convergent validity, and discrimination were confirmed. For all these reasons, it can be affirmed that the instrument used for intergenerational joint action is reliable and valid.

### 2.3. Procedure

Participants were recruited and completed the online questionnaire via the professional survey website Survey Monkey (Spain) during October 2017 [23]. The maximum time required to complete the questionnaire was 25–30 min. Once the questionnaires were completed, the data were extracted in Excel format and codified appropriately for statistical analyzes.

### 2.4. Data and Statistical Analysis

A descriptive data analysis was performed using SPSS 24.0 (IBM, Armonk, NY, USA). To describe the qualitative variables, we used frequencies and percentages. For the quantitative variables, a Shapiro–Wilk test was conducted to examine the normality of data, and central position statistics (mean or median), and measures of dispersion (standard deviation, SD; or interquartile range, IQR) were calculated. Correlations between qualitative variables were assessed using a chi-square contingency table analysis.

When participants did not complete all items in the questionnaire, missing values were resolved by removing pairs of data from the analysis. The significance level (α risk) was set up at 5% (α = 0.05).

## 3. Results

### 3.1. Sociodemographic Analysis

The analysis of the sociodemographic data of the respondents are shown in Table 2. A total of 30.2% (*n* = 608) was male, and 69.8% (*n* = 1405) was female. The mean and median age of participants was 33.96 and 26.00 years, respectively (SD = 16.01; min = 10 and max = 85 years, IQR = 25). The participants who stated performing virtual activities using virtual communication tools with people of different generations were 44.5% (*n* = 895), 10.2% (*n* = 205) using virtual educational tools, 11.9% (*n* = 239) web browser, and 20.7% (*n* = 417) social networks. 

We examined the associations between the performance of intergenerational activities using each type of virtual tool and the sociodemographic variables of the participants in the survey. The results of Pearson’s chi-square analysis are provided in Table 3. Regarding their age, most of the participants who performed intergenerational virtual activities were between 22 and 39 years old. A significant association was found between the age of the respondents and the use of social networks with people of a different generation. Differences between sex were also observed. The frequency of participants who performed intergenerational activities using virtual communication tools or social networks (Table 3) was significantly higher among women than among men (Figure S1).

Intergenerational interactions derived from the use of social networks were also associated with the place of origin, educational level, marital status, living arrangements, employment situation, and income level of the participants in the study (Table 3, Figures S1–S3). Thus, participants from an urban area, with a higher educational level, single or married, living with a partner and/or other relatives, unemployed or employed, and with a higher income level reported using social networks with people of other generation with a significantly higher frequency than the other groups of each variable.

In relation to the performance of intergenerational activities using virtual communication tools, a significant association with the living arrangements of respondents were found, with people living with a partner and/or other relatives reported more frequently (Table 3, Figure S3). In addition, participants with personal autonomy used virtual communication tools more frequently than those who needed family, professional or other support, which, although not significant, tended to be significant (*p* = 0.053). No significant differences were observed between sociodemographic groups in relation to the use of virtual educational tools and web browsers (Table 3) during activities performed with people of other generations.

### 3.2. Benefits of Performing Intergenerational Virtual Activities

The analysis of the benefits of performing virtual activities with people of other generations among the respondents of our survey is present in Table 4, Figure S4. Most participants agreed that using virtual communication tools with people of other generations produced benefits in their relationships (84.4%), social participation (73.8%), mood (77.1%), and mental health (61.2%). Regarding the use of virtual educational tools in intergenerational activities, the majority of the participants agreed that it was beneficial for their academic education (76.4%), social participation (63.7%), relationships (60.4%), mood (52.2%), professional well-being (57.1%), and self-determination (50.4%).

The respondents who used web browsers with people of a different generation reported benefits in their relationships (67.5%), academic education (65.5), social participation (64.1%), mood (55.3%), mental health (51.9%). In relation to the use of social networks during intergenerational activities, most participants believed it produced benefits in their relationships (78.6%), social participation (78.1%), mood (72.4%), and mental health (58.4%).

**Figure 1:**
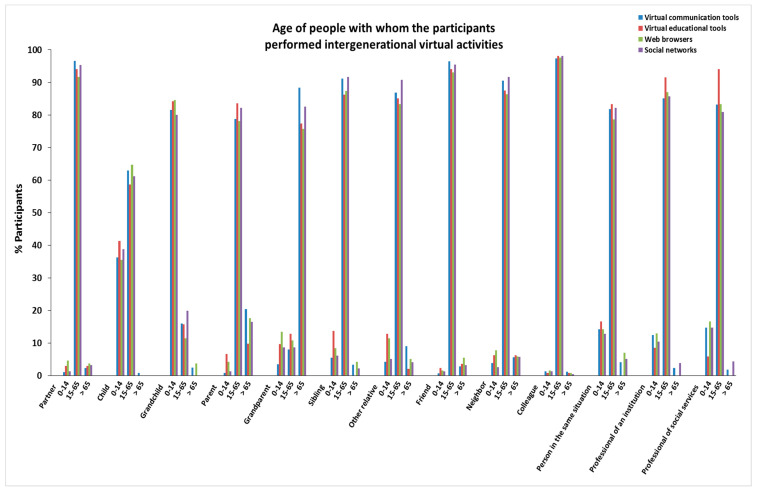
Age of people with whom the participants performed intergenerational virtual activities.

**Figure 2:**
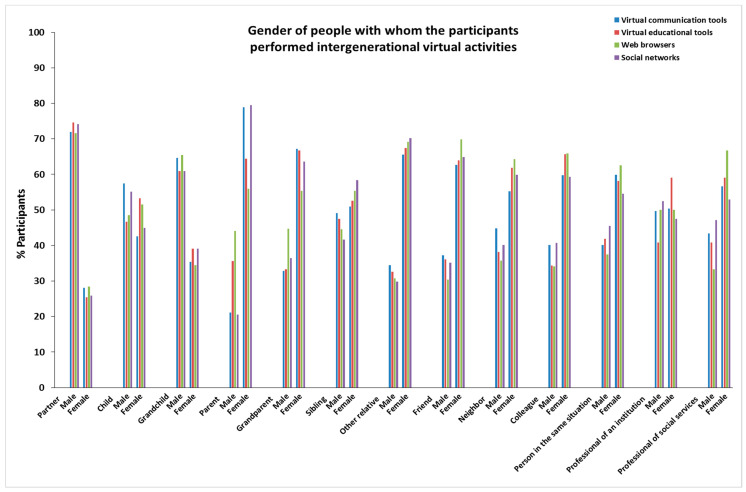
Sex of people with whom the participants performed intergenerational virtual activities.

**Figure 3:**
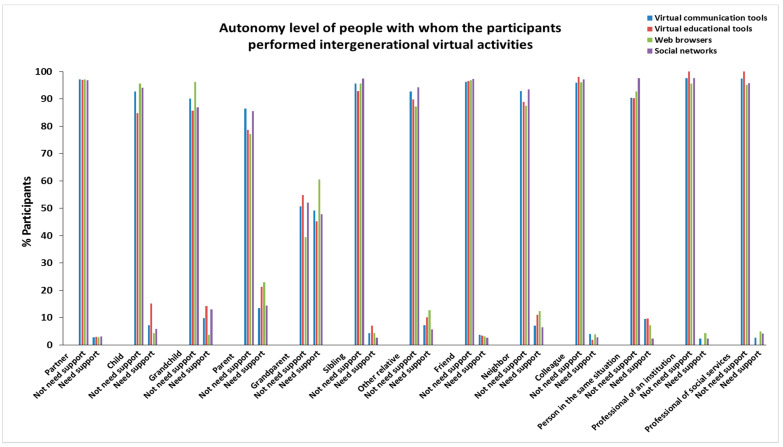
Autonomy level of people with whom the participants performed intergenerational virtual activities.

**Figure 4:**
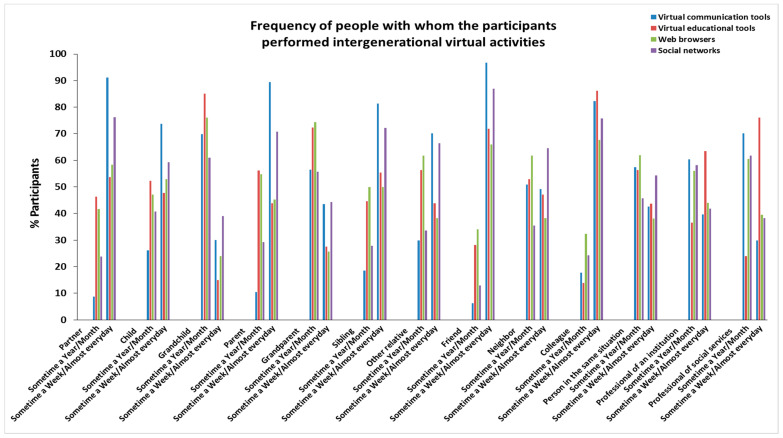
Frequency of people with whom the participants performed intergenerational virtual activities.

The characteristics of age, sex, personal autonomy, and frequency of people with whom the participants in this study performed virtual activities are provided in Figure 1, Table S1 (age); Figure 2, Table S2 (sex); Figure 3, Table S3 (personal autonomy) and Figure 4, Table S4 (frequency). No significant differences based on the type of relationship (relatives, friends, acquaintances, or professionals) of the people were found.

### 3.3. Satisfaction of Performing Intergenerational Virtual Activities

The participants in our survey were asked about the satisfaction they felt from performing virtual activities with people of other generations (Table 5, Figure S5). Considering the use of virtual communication tools, most participants were quite or very satisfied with the person with whom they performed these activities. The person more frequent reported was a friend (89.9%), followed by the partner (84.0%), sibling (83.1%), parent (81.5%), other relative (81.1%), grandparent (78.6%), colleague (79.5%), child (77.7%), and a neighbor/ acquaintance (74.0%) of the respondent. Similarly, participants were quite or very satisfied from using virtual educational tools with a sibling (83.9%), partner (83.8%), friend (80.2%), neighbor (79.4%), parent (78.0%), child (76.8%), grandparent (77.8%), other relative (76.0%), professional of health (72.1%), and colleague (71.1%). In relation to the use of web browsers or social networks, people with whom the participants were quite or very satisfied more frequently were a friend (80.5% and 83.7%, respectively), partner (75.9% and 81.6%), sibling (77.9% and 77.2%), colleague (77.8% and 74.5%), other relative (74.4% and 76.6%), and child (74.1% and 68.4%).

### 3.4. Limitations of People Who Perform Intergenerational Virtual Activities

As personal limitations or disabilities could condition the performance of intergenerational virtual activities, we asked the participants in this study if the people with whom they performed these activities had any limitations. Limitations include visual, hearing, psychic, motor, learning, behavioral, communicational, and other disabilities, autism spectrum, and attention deficit disorders. Most of the respondents reported that the person with whom they used virtual tools had no limitations or disabilities (Table 6, Figure S6). Among the people who had any limitation, they were more frequently a grandparent, with a frequency of 35.0%, 46.9%, 50.0%, and 37.8% for using virtual communicational tools, virtual educational tools, web browsers, and social networks, respectively.

## 4. Discussion

Today, a widening social gap between people of different generations is well recognized in developed societies. As a consequence, there is growing research in the field of intergenerational interactions and the benefits of programs in which people of different ages perform activities together. In these programs, ICTs can be useful virtual tools to promote intergenerational interactions and improve the quality of life and well-being of people involved. In this study, an electronic survey was used to assess the benefits, satisfaction, and limitations derived from the use of virtual tools during activities performed with people of different generations. Participants included were people living in Spain of any age and belonging to different social groups.

Digital technology, such as web browsers or social networks, has become necessary to carry out a wide variety of activities of daily life in people of all ages and, therefore, it is crucial for autonomous living and active participation in current society [17]. The use of everyday ICTs, which facilitate the capture, storage, and exchange of information, is increasing worldwide [24]. Among older people, more and more people use the Internet and virtual tools in their daily activities. In Spain, according to data from the National Institute of Statistics [25], 38.1% and 33.9% of people between 65 and 74 years old have ever used a computer and the Internet, respectively. Our results show that the ICTs more commonly used together with people of a different generation among the survey participants were the virtual communication tools (44.5%), followed by social networks (20.7%), web browsers (11.9%), and, in last place, virtual educational tools (10.2%).

We analyzed the associations between the sociodemographic characteristics of the respondents and their participation in intergenerational virtual activities. When we considered the use of social networks, significant associations were observed for all sociodemographic variables, except for the level of autonomy. Thus, intergenerational interactions through social networks were significantly more frequent among young adults, women, people from urban areas, with a higher educational level, single or married, living with a partner and/or other relatives, not retired, and with a higher income level. Activities using virtual communication tools between people of different generations were only associated with the sex and the living arrangements of the participants. No significant differences were found in relation to the use of virtual educational tools or web browsers. These results suggest that the effectiveness of intergenerational programs may be influenced by the sociodemographic characteristics of the participants when they include the use of social networks more than other virtual tools.

Many authors have reported the benefits of everyday ICTs on the well-being of older people. Chen and Schulz [26] conducted a systematic review that explored the effects of ICT interventions on reducing social isolation of the elderly. Their results suggest that ICTs could be an effective tool to tackle social isolation among the elderly. In addition, greater everyday ICT engagement predicted more positive self-perceptions of aging-related to personal competence [27]. Moreover, limitations of health conditions, the cost and difficulty of transportation, and social isolation can create barriers to travel. ICTs are also an effective communication method to use when travel is not possible [28].

In relation to intergenerational programs, which have been demonstrated to produce benefits for all the people involved [29,30], ICTs and virtual tools seem to be useful to improve interactions between people of different generations and their social participation. The use of digital technologies can enhance social connectedness across generations [31]. Chonody and Wang [32] conducted a reminiscence program for older adults aimed at intergenerational connection through multimedia. They found that media and technology were powerful ways to convey messages, and these messages combated ageism and stereotypes commonly associated with older adults (for example, that they are depressed with nothing to do), promoting a better understanding of the life course. In the Israeli Multigenerational Connection Program [33], seniors and children in computer-room activities at primary schools were encouraged to benefit by learning from each other. For older adults, the effects of this intergenerational program included increased self-esteem and the ability to participate fully in society. Children had more positive attitudes toward older people and understood them better after the program. LoBuono et al. [34] examined qualitative data from an intergenerational service-learning program in which students in higher education assisted and mentored older adults with and about technology. The authors concluded that older adults are interested in learning the technology basics, which may lead to use of technology for social, civic, and productive engagement purposes in addition to managing their health. Another study described the Engaging Generations Program, an intergenerational service-learning program that used reverse mentoring within higher education, at a public university in New England where students helped older adults learn about technology, and students gained communication and teaching skills [35]. Analysis of pre/post surveys found that students’ attitudes toward aging and older adults’ interest in technology significantly improved after program participation.

In our study, most of the participants reported that using virtual tools during intergenerational activities reported improvements in their social participation, relationships, mood, mental health, and academic education. Moreover, most participants were quite or very satisfied with the person with whom they performed intergenerational virtual activities, more frequently if this person was a friend, their partner, a sibling, another relative, or a colleague than institutional or health professionals. Finally, the people who participated in intergenerational virtual activities and who had no limitations or disabilities were more frequently reported by the respondents of the survey, except when the person was their grandparent.

This study presents several limitations that must be taken into consideration. The sample bias could affect the results. As voluntary sampling was used, factors such as motivation to complete the questionnaire, the availability of technological resources, or the level of digital competence of the participants may have influenced the final sample obtained. Moreover, if the sociodemographic, educational, and economic characteristics of the study sample were conditioned by this issue, the generalizability of our findings could be reduced. In addition, subjects who were not independent in their activities of daily living were not included in the study due to their lack of ability to complete the online survey. Despite these limitations, our study presents findings that can contribute to supporting the benefits of intergenerational interactions through ICTs and virtual tools.

In addition, it should be noted that the use of ICTs has become more relevant in intergenerational relationships. Likewise, the assessment of the benefits, satisfaction, and limitations of the performance of intergenerational virtual activities can be further amplified given the context of the COVID-19 pandemic and post pandemic. This research is especially relevant regarding current social, economic, and demographic changes that are the result of the ongoing pandemic, in a context where several studies are already beginning to appear, highlighting the importance of some of these aspects [20,21].

## 5. Conclusions

Our results suggest that intergenerational interactions through activities using virtual tools, such as virtual communication and educational tools, web browsers and social networks, can improve the social participation and relationships, as well as the mood and mental health, of all people involved in them. Additionally, interactions derived from the use of virtual tools may increase the satisfaction with people of different generations and reduce the physical and cognitive limitations of the subjects who participate in intergenerational virtual activities.

These results have a huge implication, mainly in the situation that we have to live in during the COVID-19 pandemic, which should inspire us to try to encourage participation in this type of activity in different age groups, but mainly in older people, who are the ones who a priori may have greater difficulty in using it due to their prior ignorance in virtual activities.

## Figures and Tables

**Table 1:** Original coding and recoding of the answers of participants about the benefits of performing virtual activities using (1), (2), (3), or (4), on several categories: (A). Benefit; (B). People with whom and frequency; (C). Age; (D). Sex; (E) Autonomy; (F). Limitations; and (G). Satisfaction with whom they carry out those activities.

(A). Using (1)/(2)/(3)/(4) with people of another generation produces BENEFITS for your: Physical health, Mental health, Mood, -Relationships, Self-determination, Social participation, Economic well-being, Professional well-being, Academic education. RC: Totally disagree–Disagree–Rather disagree–Neither agree nor disagree–Rather agree–Agree–Totally agree. RR: Disagree–Neither agree nor disagree–Agree.
(B). With WHO and FREQUENCY do you use (1)/(2)/(3)/(4)? Partner, Child, Grandchild, Parent, Grandparent, Sibling, Other relative, Friend, Neighbor, Colleague, Person in the same situation, Professional of an institution, Professional of health, social or academic services. RC: Sometime a year–Sometime a month–Sometime a week–Every day or almost every day. RR: Sometime a year/month–Sometime a week/Every day or almost every day.
(C). AGE of the people with whom you use (1)/(2)/(3)/(4): Partner, Child, Grandchild, Parent, Grandparent, Sibling, Other relative, Friend, Neighbor, Colleague, Person in the same situation, Professional of an institution, Professional of health, social or academic services. RC: <6–6/14–15/20–21/39–40/59–60/65–66/70–71–75–>75 years old. RR: 0/14–15/65–>65 years old.
(D). SEX of the people with whom you use (1)/(2)/(3)/(4): Partner, Child, Grandchild, Parent, Grandparent, Sibling, Other relative, Friend, Neighbor, Colleague, Person in the same situation, Professional of an institution, Professional of health, social or academic services. RC and RR: Male–Female.
(E). AUTONOMY of the people with whom you use (1)/(2)/(3)/(4): Partner, Child, Grandchild, Parent, Grandparent, Sibling, Other relative, Friend, Neighbor, Colleague, Person in the same situation, Professional of an institution, Professional of health, social or academic services. RC: Not need support–Need family–Need professional–Need other support. RR: Not need support–Need support.
(F). LIMITATION of the people with whom you use (1)/(2)/(3)/(4): Partner, Child, Grandchild, Parent, Grandparent, Sibling, Other relative, Friend, Neighbor, Colleague, Person in the same situation, Professional of an institution, Professional of health, social or academic services. RC: Without disability–Visual disability–Hearing disability–Psychic disability–Motor disability–Learning disability–Behavioral disability–Communication disability–Autism spectrum disorders–Attention deficit disorders–Others. RR: No limitation–Any limitation.
(G). SATISFACTION you feel from using (1)/(2)/(3)/(4) with these people: Partner, Child, Grandchild, Parent, Grandparent, Sibling, Other relative, Friend, Neighbor, Colleague, Person in the same situation, Professional of an institution, Professional of health, social or academic services. RC: Not satisfied at all–Little–Somewhat–Quite–Very satisfied. RR: Not/little satisfied–Neither satisfied nor dissatisfied–Quite/very satisfied.

NOTE: (1) = virtual communication tools, (2) = virtual educational tools, (3) = web browsers, and (4) = social networks. RC = response categories. RR = recategorization of responses.

**Table 2:** Sociodemographic characteristics of the participants in the study.

Variables	N (%)
Age (years)	33.96 ^1^ (16.01) ^2^
Sex Male	608 (30.2)
Female	1405 (69.8)
Birthplace	
Rural area, small village	440 (21.9)
Rural area, large village	326 (16.2)
Urban area, small town	906 (45.0)
Urban area, large town	341 (16.9)
Education	
Primary school	20 (1.0)
High school	210 (10.4)
Vocational training	138 (6.9)
College or university	1645 (81.7)
Autonomy level	
Alone	1705 (84.7)
Family support	253 (12.6)
Professional support	12 (0.6)
Other support	43 (2.1)
Marital status	
Single	1024 (50.9)
Married or in union	761 (37.8)
Widowed	23 (1.1)
Separated	25 (1.2)
Divorced	56 (2.8)
Living arrangements	
Living alone	205 (10.2)
Living with a partner	332 (16.5)
Living with a partner and children	342 (17.0)
Living with a partner and grandchildren	3 (0.1)
Living with a partner, children, and grandchildren	5 (0.2)
Living with children	36 (1.8)
Living with children and grandchildren	3 (0.1)
Living with parents	562 (27.9)
Living with grandparents	11 (0.5)
Living with parents and grandparents	43 (2.1)
Living with other relatives	43 (2.1)
Living with friends	248 (12.3)
Other types	180 (8.9)
Employment situation	
Unemployed	938 (46.6)
Employed	913 (45.4)
Retired	151 (7.5)
Income level (EUR/month)	
>2500	862 (42.8)
2001–2500	213 (10.6)
1501–2000	264 (13.1)
1001–1500	229 (11.4)
501–1000	204 (10.1)
<500	116 (5.8)

NOTE: N = number of participants, % = percentage ^1^: mean ^2^: standard deviation.

**Table 3:** Association between performing virtual activities with people of different generations and the sociodemographic characteristics of the participants.

Variables	Virtual Communication Tools (N = 895)			Virtual Educational Tools (N = 205)			Web Browsers (N = 239)			Social Networks (N = 417)		
	N (%)	χ^2^	*p*	N (%)	χ^2^	*p*	N (%)	χ^2^	*p*	N (%)	χ^2^	*p*
Age (years)												
<22 22–39 ≥40	222 (25.1) 378 (42.3) 292 (32.7)	5.055	0.088	58 (28.3) 81 (39.5) 66 (32.2)	3.800	0.150	54 (22.6)	1.294	0.524	123 (29.5)	14.015	0.001
							94 (39.3)			167 (40.0)		
							91 (38.1)			127 (30.5)		
Sex												
Male Female	259 (28.9) 636 (71.1)	8.466	0.004	58 (28.3) 147 (71.7)	1.858	0.173	70 (29.3)	1.513	2.19	110 (26.4)	10.034	0.002
							169 (70.7)			307 (73.6)		
Place of origin												
Rural area Urban area	157 (36.4) 274 (63.6)	0.94	0.332	82 (40.0) 123 (60.0)	1.447	0.229	90 (37.7)	0.139	0.709	168 (40.3)	4.308	0.038
							149 (70.3)			249 (59.7)		
Education												
Less than college or university College or university	157 (17.5) 738 (82.5)	2.604	0.107	36 (17.6) 169 (82.4)	2.158	0.142	39 (16.3)	0.672	0.412	79 (18.9)	6.524	0.011
							200 (83.7)			338 (81.1)		
Autonomy level												
Alone Family/profesional/other support	868 (97.0) 27 (3.0)	3.754	0.053	201 (98.0) 4 (2.0)	0.037	0.848	235 (98.3)	0.210	0.647	409 (98.1)	0.182	0.670
							4 (1.7)			8 (1.9)		
Marital status												
Single Married or in union Widowed/separated/divorced	433 (51.5) 359 (42.7) 49 (5.8)	3.474	0.176	106 (57.0) 68 (36.6) 12 (6.5)	5.869	0.053	113 (49.8)	3.299	0.192	219 (55.4)	11.420	0.003
							99 (43.6)			149 (37.7)		
							15 (6.6)			27 (6.8)		
Living arrangements												
Living alone/with children/with grandchildren Living with a partner/a partner and children and/or grandchildren Living with parents and/or grandparents/ other relatives Living with friends/other types	114 (12.7) 325 (36.3) 258 (28.8) 198 (22.1)	9.560	0.023	28 (13.7) 69 (33.7) 60 (29.3) 48 (23.4)	5.258	0.154	26 (10.9)	4.813	0.186	51 (12.2)	12.625	0.006
							100 (41.8)			136 (32.6)		
							62 (25.9)			132 (31.7)		
							51 (21.3)			98 (23.5)		
Employment situation												
Unemployed Employed Retired	415 (46.4) 411 (45.9) 69 (7.7)	2.607	0.272	107 (52.2) 87 (42.4) 11 (5.4)	3.632	0.163	99 (41.4)	3.057	0.217	212 (50.8)	9.527	0.009
							119 (49.8)			173 (41.5)		
							21 (8.8)			32 (7.7)		
Income level (€/month)												
>2001 1001–2001 <1000	514 (57.4) 228 (25.5) 153 (17.1)	0.660	0.719	124 (60.5) 45 (22.0) 36 (17.6)	1.854	0.396	131 (54.8)	0.292	0.864	256 (61.4)	9.878	0.007
							65 (27.2)			105 (25.2)		
							43 (18.0)			56 (13.4)		

NOTE: N = number of participants, χ^2^ = chi-square test, *p* = significance, α-risk = 0.05.

**Table 4:** Benefits reported by participants who performed intergenerational virtual activities.

	Virtual Communication Tools N (%)			Virtual Educational Tools N (%)			Web Browsers N (%)			Social Networks N (%)		
	Disagree	NA/ND	Agree	Disagree	NA/ND	Agree	Disagree	NA/ND	Agree	Disagree	NA/ND	Agree
Physical health	181 (25.5)	349 (49.1)	181 (25.5)	52 (28.6)	84 (46.2)	46 (25.3)	61 (29.6)	88 (42.7)	57 (27.7)	97 (27.6)	161 (45.9)	93 (26.5)
Mental health	62 (8.7)	214 (30.1)	435 (61.2)	20 (11.0)	73 (40.7)	89 (48.9)	28 (13.6)	71 (34.5)	107 (51.9)	46 (13.1)	100 (28.5)	205 (58.4)
Mood	35 (4.9)	128 (18.0)	548 (77.1)	22 (12.1)	65 (35.7)	95 (52.2)	24 (11.7)	68 (33.0)	114 (55.3)	29 (8.3)	68 (19.4)	254 (72.4)
Relationships	30 (4.2)	81 (11.4)	600 (84.4)	18 (9.9)	54 (29.7)	110 (60.4)	15 (7.3)	52 (25.2)	139 (67.5)	19 (5.4)	56 (16.0)	276 (78.6)
Self- determination	99 (13.9)	311 (43.7)	301 (42.3)	23 (12.6)	67 (36.8)	92 (50.5)	24 (11.7)	79 (38.3)	103 (50.0)	56 (16.0)	151 (43.0)	144 (41.0)
Social participation	42 (5.9)	144 (20.3)	525 (73.8)	17 (9.2)	49 (26.9)	116 (63.7)	17 (8.3)	57 (27.7)	132 (64.1)	17 (4.8)	60 (17.1)	274 (78.1)
Economic well-being	198 (27.8)	374 (52.6)	139 (19.5)	41 (22.5)	86 (47.3)	55 (30.2)	58 (28.2)	98 (47.6)	50 (24.3)	110 (31.3)	177 (50.4)	64 (18.2)
Professional well-being	138 (19.4)	316 (44.4)	257 (36.1)	24 (13.2)	54 (29.7)	104 (57.1)	28 (13.6)	73 (35.4)	105 (51.0)	81 (23.1)	154 (43.9)	116 (33.0)
Academic education	83 (11.7)	280 (39.4)	348 (48.9)	10 (5.5)	33 (18.1)	139 (76.4)	17 (8.3)	54 (26.2)	135 (65.5)	56 (16.0)	127 (36.2)	168 (47.9)

NOTE: N = number of participants, % = percentage, NA/ND = neither agree nor disagree.

**Table 5:** Level of satisfaction reported by participants who performed intergenerational virtual activities.

	Virtual Communication Tools N (%)			Virtual Educational Tools N (%)			Web Browsers N (%)			Social Networks N (%)		
	Not/Little Satisfied	NA/ ND	Quite/ Very Satisfied	Not/ Little Satisfied	NA/ ND	Quite/ Very Satisfied	Not/ Little Satisfied	NA/ ND	Quite/ Very Satisfied	Not/ Little Satisfied	NA/ ND	Quite/ Very Satisfied
Partner	17 (3.6)	58 (12.4)	394 (84.0)	5 (7.4)	6 (8.8)	57 (83.8)	10 (8.6)	18 (15.5)	88 (75.9)	14 (6.5)	26 (12.0)	177 (81.6)
Child	30 (12.9)	22 (9.4)	181 (77.7)	11 (13.4)	8 (9.8)	63 (76.8)	12 (14.8)	9 (11.1)	60 (74.1)	15 (15.3)	16 (16.3)	67 (68.4)
Grandchild	27 (31.8)	8 (9.4)	50 (58.8)	7 (31.8)	2 (9.1)	13 (59.1)	11 (25.0)	6 (13.6)	27 (61.4)	15 (31.9)	7 (14.9)	25 (53.2)
Parent	18 (3.4)	79 (15.1)	426 (81.5)	5 (8.5)	8 (13.6)	46 (78.0)	9 (9.3)	23 (23.7)	65 (67.0)	13 (6.3)	46 (22.3)	147 (71.4)
Grandparent	4 (14.3)	2 (7.1)	22 (78.6)	5 (16.1)	2 (6.5)	24 (77.4)	8 (20.0)	9 (22.5)	23 (57.5)	8 (11.3)	11 (15.5)	52 (73.2)
Sibling	19 (3.9)	63 (13.0)	404 (83.1)	5 (8.9)	4 (7.1)	47 (83.9)	7 (7.4)	14 (14.7)	74 (77.9)	12 (5.3)	40 (17.5)	176 (77.2)
Other relative	15 (3.3)	70 (15.6)	365 (81.1)	5 (10.0)	7 (14.0)	38 (76.0)	6 (7.7)	14 (17.9)	58 (74.4)	10 (4.7)	40 (18.7)	164 (76.6)
Friend	8 (1.3)	53 (8.8)	542 (89.9)	4 (4.7)	13 (15.1)	69 (80.2)	5 (3.9)	20 (15.6)	103 (80.5)	10 (3.3)	39 (13.0)	252 (83.7)
Neighbor	16 (5.1)	66 (21.0)	233 (74.0)	4 (11.8)	3 (8.8)	27 (79.4)	9 (15.8)	11 (19.3)	37 (64.9)	15 (9.1)	35 (21.2)	115 (69.7)
Colleague	15 (3.2)	82 (17.3)	376 (79.5)	9 (8.5)	21 (19.8)	76 (71.1)	6 (4.8)	22 (17.5)	98 (77.8)	6 (2.8)	48 (22.6)	158 (74.5)
Person in the same situation	21 (14.8)	27 (19.0)	94 (66.2)	6 (18.8)	4 (12.5)	22 (68.8)	11 (26.8)	4 (9.2)	26 (63.4)	12 (14.5)	19 (22.9)	52 (62.7)
Professional of an institution	22 (13.8)	41 (25.6)	97 (60.6)	8 (17.0)	6 (12.8)	33 (70.2)	9 (18.8)	9 (18.8)	30 (62.5)	13 (15.7)	24 (28.9)	46 (55.4)
Professional of social services	25 (17.0)	33 (22.4)	89 (60.5)	9 (13.2)	10 (14.7)	49 (72.1)	9 (22.5)	5 (12.5)	26 (65.0)	10 (14.3)	14 (20.0)	46 (65.7)

NOTE: N = number of participants, % = percentage, NA/ND = neither agree nor disagree.

**Table 6:** Limitations of people with whom participants performed intergenerational virtual activities.

	Virtual communication Tools N (%)		Virtual educational Tools N (%)		Web Browsers N (%)		Social Networks N (%)	
	No Limitation	Any Limitation	No Limitation	Any Limitation	No Limitation	Any Limitation	No Limitation	Any Limitation
Partner	420 (91.7)	38 (8.3)	57 (83.8)	11 (16.2)	96 (88.1)	13 (11.9)	190 (88.0)	26 (12.0)
Child	210 (87.9)	29 (12.1)	37 (80.4)	9 (19.6)	61 (88.4)	8 (11.6)	88 (85.4)	15 (14.6)
Grandchild	75 (85.2)	13 (14.8)	17 (81.0)	4 (19.0)	20 (76.9)	6 (23.1)	42 (82.4)	9 (17.6)
Parent	442 (85.0)	78 (15.0)	46 (76.7)	14 (23.3)	78 (78.8)	21 (21.2)	175 (84.1)	33 (15.9)
Grandparent	132 (65.0)	71 (35.0)	17 (53.1)	15 (46.9)	19 (50.0)	19 (50.0)	46 (62.2)	28 (37.8)
Sibling	435 (90.4)	46 (9.6)	49 (87.5)	7 (12.5)	83 (91.2)	8 (8.8)	199 (87.7)	28 (12.3)
Other relative	398 (90.2)	43 (9.8)	43 (89.6)	5 (10.4)	68 (87.2)	10 (12.8)	193 (89.4)	23 (10.6)
Friend	547 (92.6)	44 (7.4)	79 (92.9)	6 (7.1)	113 (89.7)	13 (10.3)	275 (91.7)	25 (8.3)
Neighbor	287 (90.0)	32 (10.0)	27 (79.4)	7 (20.6)	44 (80.0)	11 (20.0)	144 (84.7)	26 (15.3)
Colleague	423 (92.6)	34 (7.4)	99 (92.5)	8 (7.5)	116 (92.8)	9 (7.2)	190 (90.9)	19 (9.1)
Person in the same situation	127 (84.1)	24 (15.9)	27 (84.4)	5 (15.6)	33 (78.6)	9 (21.4)	70 (85.4)	12 (14.6)
Professional of an institution	139 (86.9)	21 (13.1)	43 (91.5)	4 (8.5)	36 (76.6)	11 (23.4)	70 (86.4)	11 (13.6)
Professional of social services	130 (87.8)	18 (12.2)	9 (100.0)	0 (0.0)	32 (80.0)	8 (20.0)	62 (84.9)	11 (15.1)

NOTE: N = number of participants, % = percentage.

## Data Availability

The data sets generated for this study are available on request to the corresponding author.

## Supplementary Materials

The following are available online at www.mdpi.com/article/10.3390/ijerph19010401/s1, Figure S1: Association between virtual intergenerational activities and the sociodemographic characteristics of the participants: Age, Gender and Birthplace; Figure S2: Association between virtual intergenerational activities and the sociodemographic characteristics of the participants: Education, Autonomy level and Marital status; Figure S3: Association between virtual intergenerational activities and the sociodemographic characteristics of the participants: Living arrangement, Employment situation and Income level (€/month); Figure S4: Benefits reported by participants who performed intergenerational virtual activities; Figure S5: Level of satisfaction reported by participants who performed intergenerational virtual activities; Figure S6: Limitations of people with whom participants performed intergenerational virtual activities; Table S1: Age of people with whom the participants performed intergenerational virtual activities; Table S2: Gender of people with whom the participants performed intergenerational virtual activities; Table S3: Autonomy level of people with whom the participants performed intergenerational virtual activities; Table S4: Frequency of people with whom the participants performed intergenerational virtual activities.

## References

[1] Wang H. (2020). GBD 2019 Demographics Collaborators. Global age-sex-specific fertility, mortality, healthy life expectancy (HALE), and population estimates in 204 countries and territories, 1950–2019: A comprehensive demographic analysis for the Global Burden of Disease Study 2019. Lancet.

[2] WHO (2020). Decade of Healthy Ageing: Baseline Report Geneva.

[3] Wilson C.A., Saklofske D.H. (2018). The relationship between trait emotional intelligence, resiliency, and mental health in older adults: The mediating role of savouring. Aging Ment. Health..

[4] Czaja S.J., Moxley J.H., Rogers W.A. (2021). Social Support, Isolation, Loneliness, and Health among Older Adults in the PRISM Randomized Controlled Trial. Front. Psychol..

[5] Armitage R., Nellums L.B. (2020). COVID-19 and the consequences of isolating the elderly. Lancet Public Health..

[6] Wu B. (2020). Social isolation and loneliness among older adults in the context of COVID-19: A global challenge. Global Health Res. Policy..

[7] Mickus M.A., Luz C.C. (2002). Televisits: Sustaining long distance family relationships among institutionalized elders through technology. Aging Ment. Health.

[8] Smith A. (2014). Older Adults and Technology Use.

[9] Marston H.R., Kroll M., Fink D., de Rosario H., Gschwind Y.J. (2016). Technology use, adoption and behavior in older adults: Results from the iStoppFalls project. Educ. Gerontol..

[10] Marston H.R., Samuels J. (2019). A review of age friendly virtual assistive technologies and their effect on daily living for careers and dependent adults. Healthcare.

[11] Celdrán M., Serrat R., Villar F., Montserrat R. (2021). Exploring the Benefits of Proactive Participation among Adults and Older People by Writing Blogs. J. Gerontol. Soc. Work..

[12] Blok M., Ingen E.J.V., de Boer A., Slootman M.W. (2020). ICT als instrument voor het sociaal en emotioneel welbevinden [ICT as an instrument for social and emotional ageing. A qualitative study with older adults with cognitive impairments]. Tijdschr. Gerontol. Geriatr..

[13] Álvarez-García J., Durán-Sánchez A., Del Río-Rama M.C., Correa-Quezada R. (2019). Older Adults and Digital Society: Scientific Coverage. Int. J. Environ. Res. Public Health..

[14] Chen W. (2013). Internet use, online communication, and ties in Americans’ networks. Soc. Sci. Comput. Rev..

[15] Lissitsa S., Chachashvili-Bolotin S. (2016). Life satisfaction in the internet age—Changes in the past decade. Comput. Hum. Behav..

[16] Hill R., Betts L.R., Gardner S.E. (2015). Older adults’ experiences and perceptions of digital technology: (Dis)empowerrment, wellbeing, and inclusion. Comput. Hum. Behav..

[17] Schmidt L.I., Wahl H.W. (2019). Predictors of performance in everyday technology tasks in older adults with and without mild cognitive impairment. Gerontologist.

[18] García-Martin J., García J.N. (2013). Patterns of Web 2.0 tool use among young Spanish people. Comput Educ..

[19] Díaz-Prieto C., García-Sánchez J.N., Canedo-García A. (2019). Impact of Life Experiences and Use of Web 2.0 Tools in Adults and Older Adults. Front. Psychol..

[20] Britton B. (2020). Case study: WhatsApp support through the COVID-19 pandemic. Nurs. Resid. Care.

[21] Schuster A.M., Cotton S.R. (2021). COVID-19’s Influence on Information and Communication Technologies in Long-Term Care: Results from an Online Survey with Long-Term Care Administrators. JMIR Aging..

[22] Canedo-García A., García-Sánchez J.N., Pacheco-Sanz D.I. (2017). A Systematic Review of the Effectiveness of Intergenerational Programs. Front. Psychol..

[23] Canedo-García A., García-Sánchez J.N., Pacheco-Sanz D.I. (2019). Acción conjunta intergeneracional (ACIG). Descripción de variables intervinientes. Int. J. Dev. Educ. Psychology. Rev. Infad Psicol..

[24] Wallcook S., Nygård L., Kottorp A., Malinowsky C. (2019). The use of everyday information communication technologies in the lives of older adults living with and without dementia in Sweden. Assist Technol..

[25] Instituto Nacional de Estadística (INE) Encuesta Sobre Equipamiento y uso de Tecnologías de Información y Comunicación en los Hogares 2015 (TIC-H’15). https://www.ine.es/metodologia/t25/t25304506615.pdf.

[26] Chen Y.R., Schulz P.J. (2016). The effect of information and communication technology interventions on reducing social isolation in the elderly: A systematic review. J. Med. Int. Res..

[27] Köttl H., Cohn-Schwartz E., Ayalon L. (2021). Self-Perceptions of Aging and Everyday ICT Engagement: A Test of Reciprocal Associations. J. Gerontol. B.

[28] Freeman S., Marston H.R., Olynick J., Musselwhite C., Kulczycki C., Genoe R., Xiong B. (2020). Intergenerational Effects on the Impacts of Technology Use in Later Life: Insights from an International, Multi-Site Study. Int. J. Environ. Res. Public Health..

[29] Yasunaga M., Murayama Y., Takahashi T., Ohba H., Suzuki H., Nonaka K., Kuraoka M., Sakurai R., Nishi M., Sakuma N. (2016). Multiple impacts of an intergenerational program in Japan: Evidence from the Research on Productivity through Intergenerational Sympathy Project. Geriatr. Gerontol. Int..

[30] Burnes D., Sheppard C., Henderson C.R., Wassel M., Cope R., Barber C., Pillemer K. (2019). Interventions to Reduce Ageism Against Older Adults: A Systematic Review and Meta-Analysis. Am. J. Publ. Health..

[31] Taipale S. (2019). Intergenerational Connections in Digital Families.

[32] Chonody J., Wang D. (2013). Connecting Older Adults to the Community through Multimedia: An Intergenerational Reminiscence Program. Activ. Adapt. Aging..

[33] Gamliel T., Gabay N. (2014). Knowledge exchange, social interactions, and empowerment in an intergenerational technology program at school. Educ. Gerontol..

[34] LoBuono D.L., Leedahl S.N., Maiocco E. (2019). Older adults learning technology in an in-tergenerational program: Qualitative analysis of areas of technology requested for assistance. Gerontechnology.

[35] Leedahl S.N., Brasher M.S., Estus E., Breck B.M., Dennis C.B., Clark S.C. (2019). Implementing an interdisciplinary intergenerational program using the Cyber Seniors^®^ reverse mentoring model within higher education. Gerontol. Geriatr. Educ..

