# Supplementary material for: Benefits, Satisfaction and Limitations Derived from the Performance of Intergenerational Virtual Activities: Data from a General Population Spanish Survey
Source: Int J Environ Res Public Health. 2021 Dec 30;19(1):401. doi: 10.3390/ijerph19010401 (PMC8744636; doi:10.3390/ijerph19010401)
Supplement: Supplementary file 1 [file ijerph-19-00401-s001.zip › ijerph-1513440-supplementary.pdf]

Captions Suplementary Figures

Figure S1
Association between virtual intergenerational activities and the sociodemographic characteristics of the participants: Age, Gender and Birthplace
Figure S2
Association between virtual intergenerational activities and the sociodemographic characteristics of the participants: Education, Autonomy level and Marital status
Figure S3
Association between virtual intergenerational activities and the sociodemographic characteristics of the participants: Living arrangement, Employment situation and Income level (€/month).
Figure S4
Benefits reported by participants who performed intergenerational virtual activities
Figure S5
Level of satisfaction reported by participants who performed intergenerational virtual activities
Figure S6
Limitations of people with whom participants performed intergenerational virtual activities

Captions Suplementary Tables

Table S1
Age of people with whom the participants performed intergenerational virtual activities
Table S2
Gender of people with whom the participants performed intergenerational virtual activities
Table S3
Autonomy level of people with whom the participants performed intergenerational virtual activities
Table S4
Frequency of people with whom the participants performed intergenerational virtual activities.

Figure S1

Association between virtual intergenerational activities and the sociodemographic characteristics of the participants: Age, Gender and Birthplace

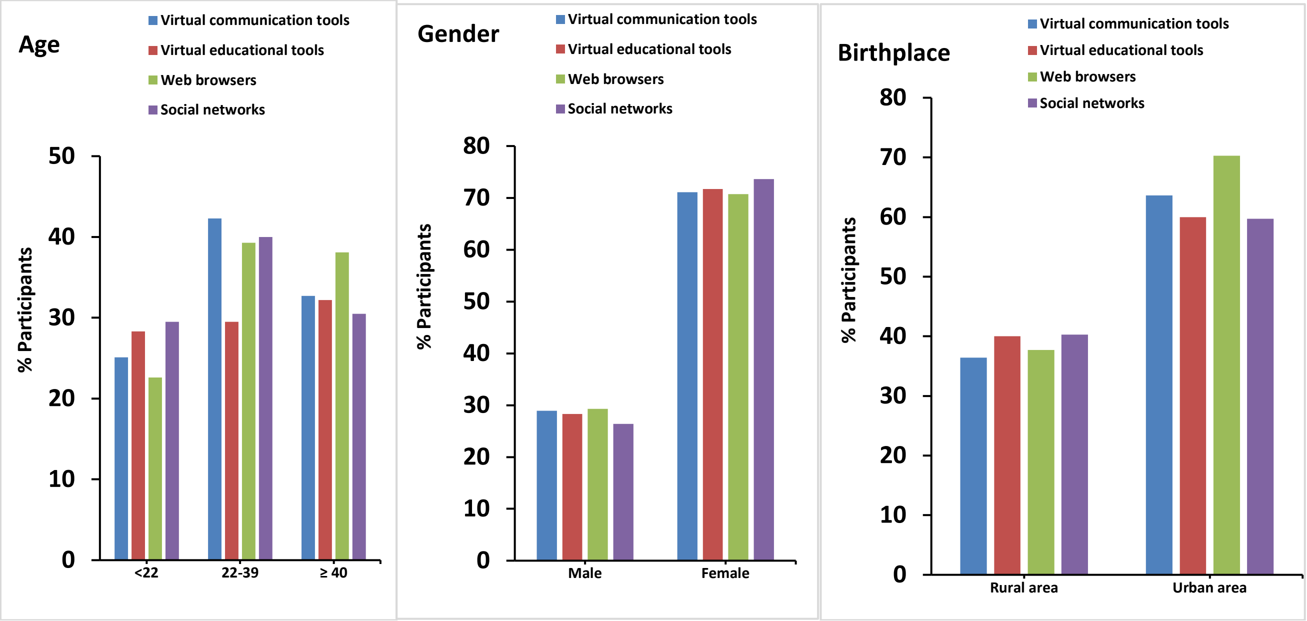

Figure S2

Association between virtual intergenerational activities and the sociodemographic characteristics of the participants: Education, Autonomy level and Marital status

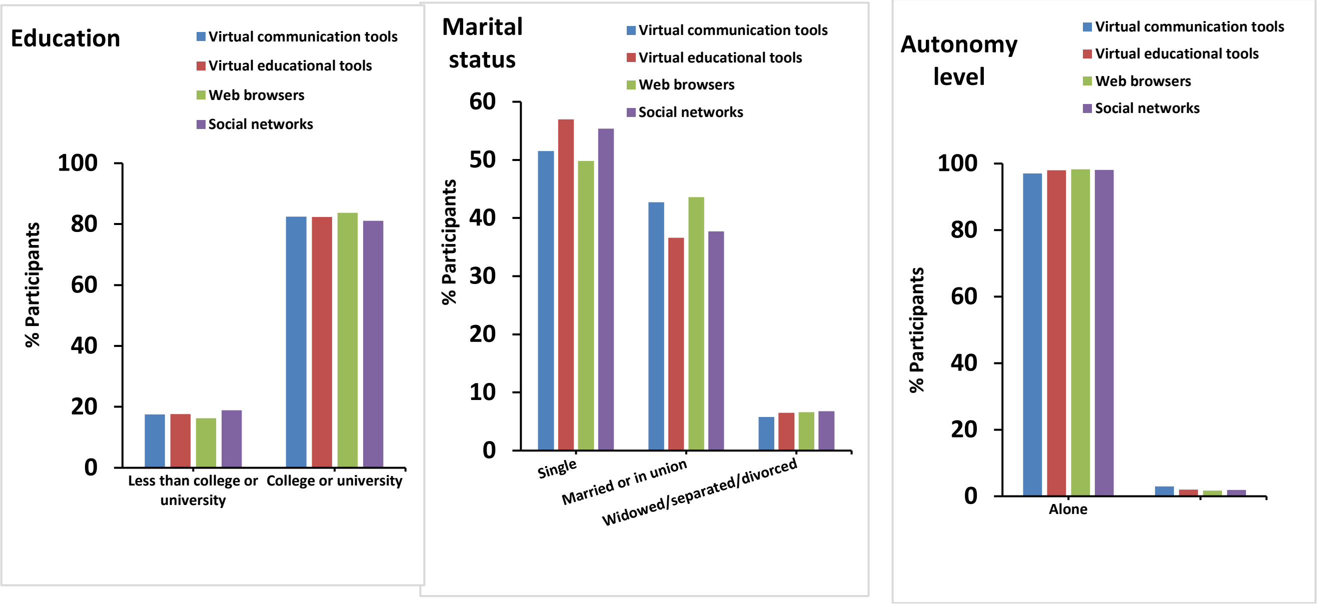

Figure S3

Association between virtual intergenerational activities and the sociodemographic characteristics of the participants: Living arrangement, Employment situation and Income level (€/month)

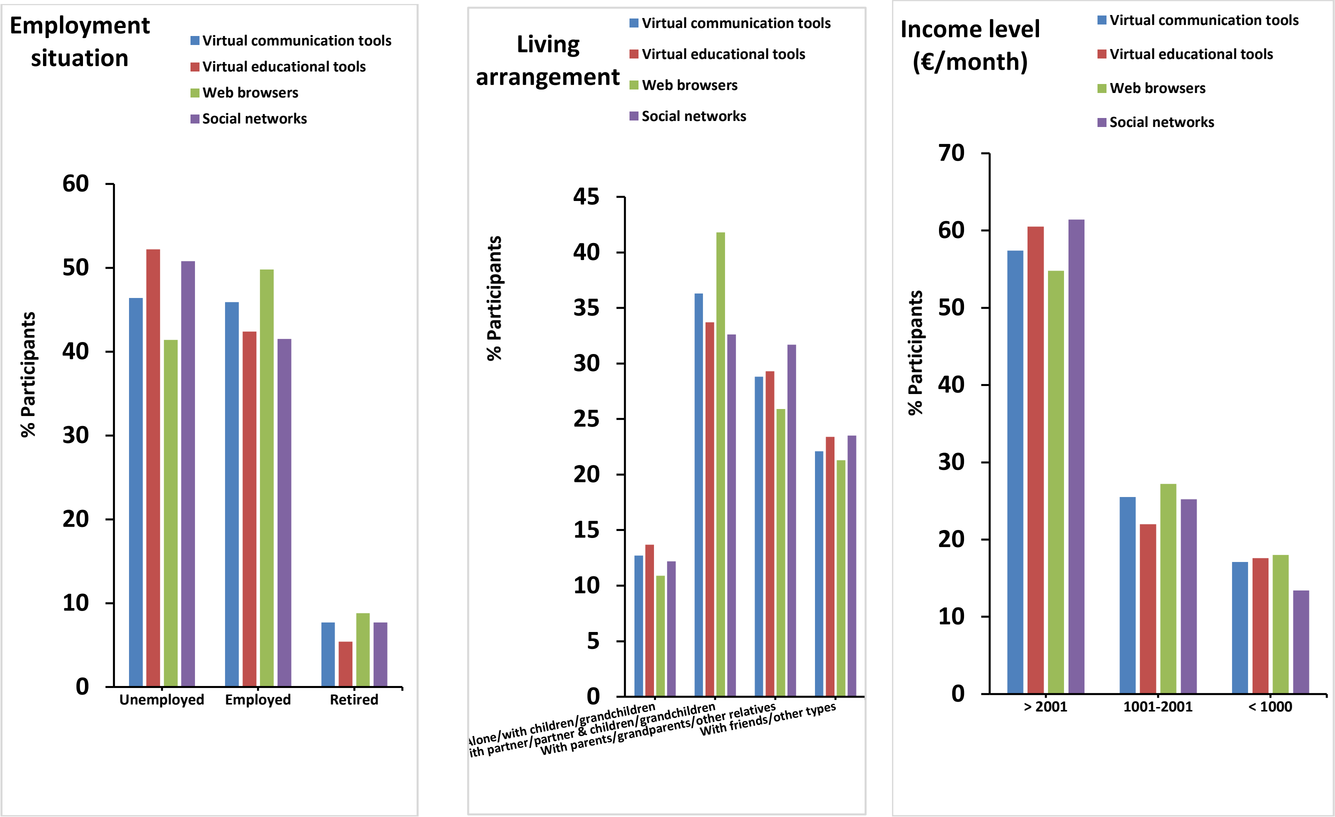

Figure S4

Benefits reported by participants who performed intergenerational virtual activities

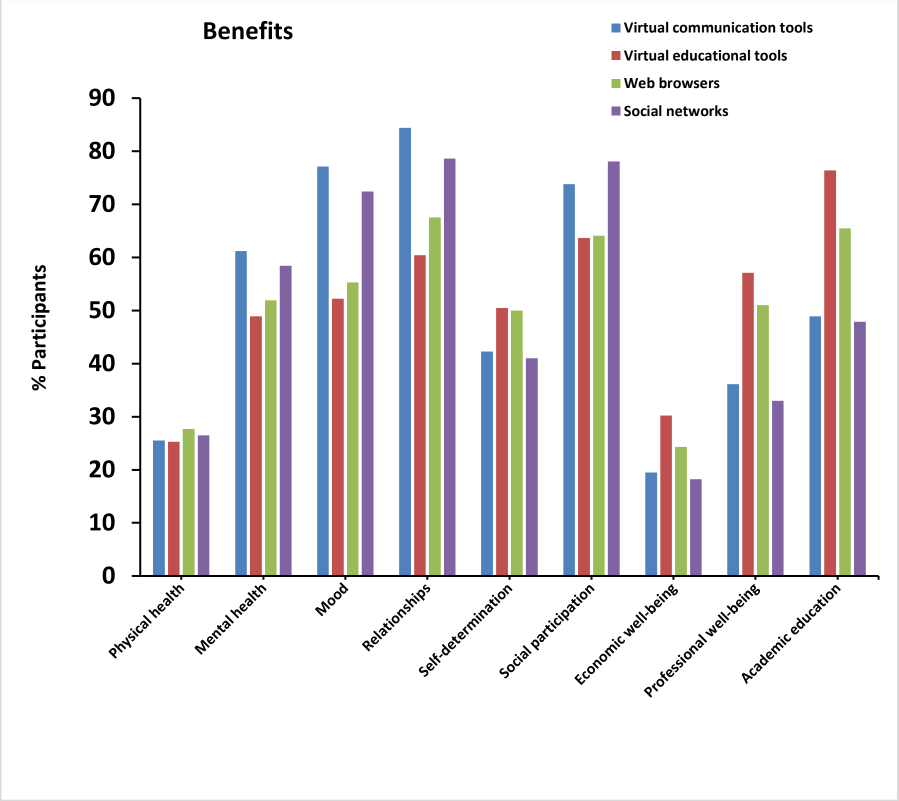

Figure S5

Level of satisfaction reported by participants who performed intergenerational virtual activities

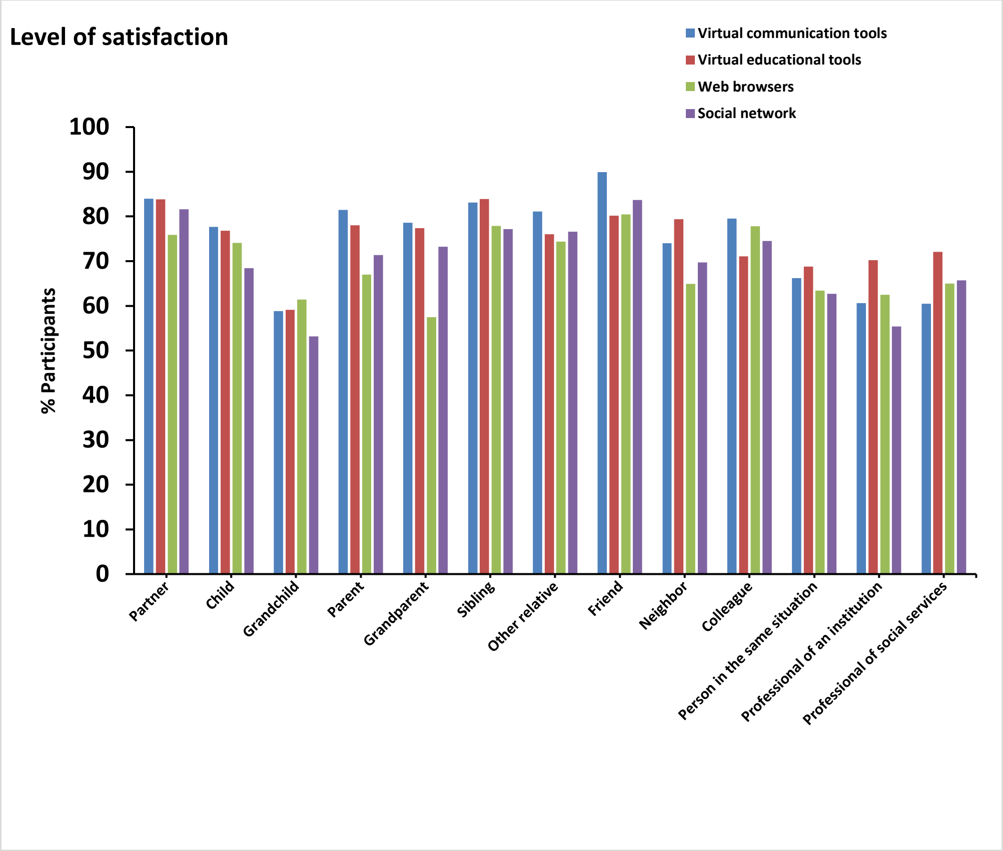

Figure S6

Limitations of people with whom participants performed intergenerational virtual activities

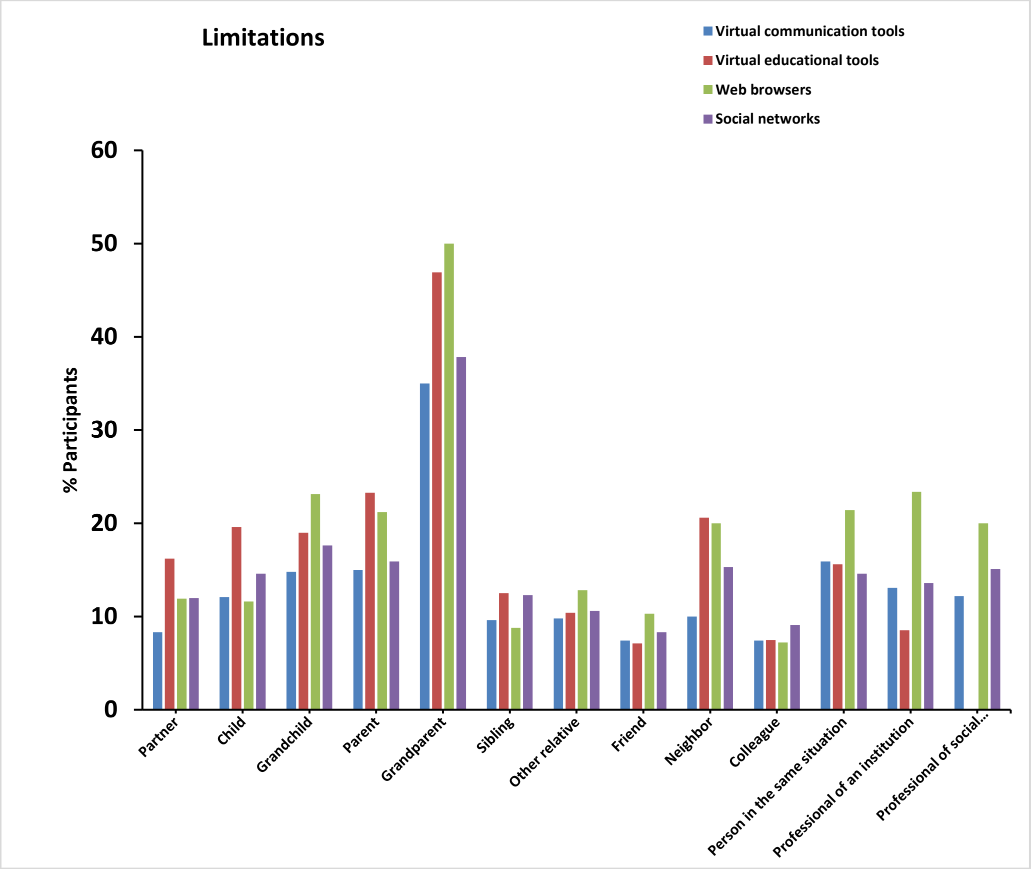

Captions Suplementary Tables

Table S1
Age of people with whom the participants performed intergenerational virtual activities
Table S2
Gender of people with whom the participants performed intergenerational virtual activities
Table S3
Autonomy level of people with whom the participants performed intergenerational virtual activities
Table S4
Frequency of people with whom the participants performed intergenerational virtual activities.

Table S1

Age of people with whom the participants performed intergenerational virtual activities

	Virtual communication tools N (%)			Virtual educational tools N (%)			Web browsers N (%)			Social networks N (%)		
	**0-14**	**15-65**	**0-14**	**0-14**	**15-65**	**> 65**	**0-14**	**15-65**	**> 65**	**0-14**	**15-65**	**> 65**
Partner	5 (1.1)	451 (96.6)	11 (2.4)	2 (3.0)	63 (94.0)	2 (3.0)	5 (4.6)	100 (91.7)	4 (3.7)	3 (1.4)	204 (95.3)	7 (3.3)
Child	84 (36.2)	146 (62.9)	2 (0.9)	19 (41.3)	27 (58.7)	0 (0)	24 (35.5)	44 (64.7)	0 (0)	38 (38.8)	60 (61.2)	0 (0)
Grandchild	66 (81.5)	13 (16.0)	2 (2.5)	16 (84.2)	3 (15.8)	0 (0)	22 (84.6)	3 (11.5)	1 (3.8)	36 (80.0)	9 (20.0)	0 (0)
Parent	4 (0.8)	412 (78.8)	107 (20.5)	4 (6.6)	51 (83.6)	6 (9.8)	4 (4.2)	75 (78.1)	17 (17.7)	3 (1.4)	174 (82.1)	35 (16.5)
Grandparent	7 (3.5)	16 (8.1)	175 (88.4)	3 (9.7)	4 (12.9)	24 (77.4)	5 (13.5)	4 (10.8)	28 (75.7)	6 (8.7)	6 (8.7)	57 (82.6)
Sibling	27 (5.5)	449 (91.1)	17 (3.4)	8 (13.8)	50 (86.2)	0 (0)	8 (8.4)	83 (87.4)	4 (4.2)	14 (6.2)	208 (91.6)	5 (2.2)
Other relative	19 (4.2)	397 (86.9)	41 (9.0)	6 (12.8)	40 (85.1)	1 (2.1)	9 (11.5)	65 (83.3)	4 (5.1)	11 (5.1)	197 (90.8)	9 (4.1)
Friend	4 (0.7)	589 (96.4)	18 (2.9)	2 (2.4)	79 (94.0)	3 (3.6)	2 (1.6)	119 (93.0)	7 (5.5)	4 (1.3)	294 (95.5)	10 (3.2)
Neighbor	12 (3.9)	275 (90.5)	17 (5.6)	2 (6.3)	28 (87.5)	2 (6.3)	4 (7.8)	44 (86.3)	3 (5.9)	4 (2.6)	143 (91.7)	9 (5.8)
Colleague	7 (1.4)	475 (97.3)	6 (1.2)	1 (0.9)	104 (98.1)	1 (0.9)	2 (1.6)	123 (97.6)	1 (0.8)	3 (1.4)	210 (98.1)	1 (0.5)
Person in the same situation	21 (14.2)	121 (81.8)	6 (4.1)	5 (16.7)	25 (83.3)	0 (0)	6 (14.3)	33 (78.6)	3 (7.1)	10 (12.8)	64 (82.1)	4 (5.1)
Professional of an institution	21 (12.5)	143 (85.1)	4 (2.4)	4 (8.5)	43 (91.5)	0 (0)	6 (13.0)	40 (87.0)	0 (0)	8 (10.4)	66 (85.7)	3 (3.9)
Professional of social services	23 (14.8)	129 (83.2)	3 (1.9)	4 (5.9)	64 (94.1)	0 (0)	6 (16.7)	30 (83.3)	0 (0)	10 (14.7)	55 (80.9)	3 (4.4)

NOTE: N=number of participants, %=percentage

Table S2

Gender of people with whom the participants performed intergenerational virtual activities

	Virtual communication tools N (%)		Virtual educational tools N (%)		Web browsers N (%)		Social networks N (%)	
	Male	Female	Male	Female	Male	Female	Male	Female
Partner	336 (71.9)	131 (28.1)	50 (74.6)	17 (25.4)	78 (71.6)	31 (28.4)	160 (74.1)	56 (25.9)
Child	132 (57.4)	98 (42.6)	21 (46.7)	24 (53.3)	33 (48.5)	35 (51.5)	54 (55.1)	44 (44.9)
Grandchild	51 (64.6)	28 (35.4)	14 (60.9)	9 (39.1)	17 (65.4)	9 (34.5)	28 (60.9)	18 (39.1)
Parent	104 (21.1)	389 (78.9)	21 (35.6)	38 (64.4)	41 (44.1)	52 (55.9)	42 (20.5)	163 (79.5)
Grandparent	63 (32.8)	129 (67.2)	10 (33.3)	20 (66.7)	17 (44.7)	21 (55.3)	24 (36.4)	42 (63.6)
Sibling	238 (49.1)	247 (50.9)	27 (47.4)	30 (52.6)	41 (44.6)	51 (55.4)	94 (41.6)	132 (58.4)
Other relative	150 (34.5)	285 (65.5)	15 (32.6)	31 (67.4)	24 (30.8)	54 (69.2)	62 (29.8)	146 (70.2)
Friend	213 (37.3)	358 (62.7)	30 (36.1)	53 (63.9)	38 (30.4)	87 (69.9)	102 (35.2)	188 (64.8)
Neighbor	130 (44.8)	160 (55.2)	13 (38.2)	21 (61.8)	20 (35.7)	36 (64.3)	65 (40.1)	97 (59.9)
Colleague	183 (40.2)	272 (59.8)	35 (34.3)	67 (65.7)	42 (34.1)	81 (65.9)	83 (40.7)	121 (59.3)
Person in the same situation	55 (40.1)	82 (59.9)	13 (41.9)	18 (58.1)	15 (37.5)	25 (62.5)	35 (45.5)	42 (54.5)
Professional of an institution	77 (49.7)	78 (50.3)	13 (40.9)	18 (59.1)	22 (50.0)	22 (50.0)	42 (52.5)	38 (47.5)
Professional of social services	63 (43.4)	82 (56.6)	27 (40.9)	39 (59.1)	13 (33.3)	26 (66.7)	32 (47.1)	36 (52.9)

NOTE: N=number of participants, %=percentage

Table S3

Autonomy level of people with whom the participants performed intergenerational virtual activities

	Virtual communication tools N (%)		Virtual educational tools N (%)		Web browsers N (%)		Social networks N (%)	
	Not NS	NS	Not NS	NS	Not NS	NS	Not NS	NS
Partner	459 (97.2)	13 (2.8)	65 (97.0)	2 (3.0)	105 (97.2)	3 (2.8)	210 (96.8)	7 (3.2)
Child	215 (92.7)	17 (7.3)	39 (84.8)	7 (15.2)	66 (95.7)	3 (4.3)	96 (94.1)	6 (5.9)
Grandchild	73 (90.1)	8 (9.9)	18 (85.7)	3 (14.3)	25 (96.2)	1 (3.8)	40 (87.0)	6 (13.0)
Parent	455 (86.5)	71 (13.5)	48 (78.7)	13 (21.3)	74 (77.1)	22 (22.9)	178 (85.6)	30 (14.4)
Grandparent	104 (50.7)	101 (49.3)	17 (54.8)	14 (45.2)	15 (39.5)	23 (60.5)	38 (52.1)	35 (47.9)
Sibling	464 (95.7)	21 (4.3)	52 (92.9)	4 (7.1)	88 (95.7)	4 (4.3)	224 (97.4)	6 (2.6)
Other relative	425 (92.8)	33 (7.2)	52 (89.8)	4 (10.2)	68 (87.2)	10 (12.8)	199 (94.3)	12 (5.7)
Friend	576 (96.2)	23 (3.8)	84 (96.6)	3 (3.4)	124 (96.9)	4 (3.1)	293 (97.3)	8 (2.7)
Neighbor	302 (92.9)	23 (7.1)	32 (88.9)	4 (11.1)	49 (87.5)	7 (12.5)	157 (93.5)	11 (6.5)
Colleague	302 (96.0)	23 (4.0)	106 (98.1)	2 (1.9)	123 (96.1)	5 (3.9)	207 (97.2)	6 (2.8)
Person in the same situation	142 (90.4)	15 (9.6)	28 (90.3)	3 (9.7)	38 (92.7)	3 (7.3)	80 (97.6)	2 (2.4)
Professional of an institution	164 (97.6)	4 (2.4)	50 (100.0)	0 (0)	44 (95.7)	2 (4.3)	80 (97.6)	2 (2.4)
Professional of social services	151 (97.4)	4 (2.6)	68 (100.0)	0 (0)	39 (95.1)	2 (4.9)	69 (95.8)	3 (4.2)

NOTE: N=number of participants, %=percentage, NS= Need support

Table S4

Frequency of people with whom the participants performed intergenerational virtual activities

	**Virtual communication tools N (%)**		**Virtual educational tools N (%)**		**Web browsers N (%)**		**Social networks N (%)**	
	Sometime a Y/M	Sometime a W/AEd	Sometime a Y/M	Sometime a W/AEd	Sometime a Y/M	Sometime a W/AEd	Sometime a Y/M	Sometime a W/AEd
Partner	41 (8.8)	425 (91.2)	31 (46.3)	36 (53.7)	45 (41.7)	63 (58.3)	50 (23.8)	160 (76.2)
Child	61 (26.2)	172 (73.8)	24 (52.2)	22 (47.8)	32 (47.1)	36 (52.9)	40 (40.8)	58 (59.2)
Grandchild	58 (69.9)	25 (30.1)	17 (85.0)	3 (15.0)	19 (76.0)	6 (24.0)	28 (60.9)	18 (39.1)
Parent	55 (10.5)	468 (89.5)	32 (56.1)	25 (43.9)	52 (54.7)	43 (45.3)	61 (29.3)	147 (70.7)
Grandparent	110 (56.4)	85 (43.6)	21 (72.4)	8 (27.6)	26 (74.3)	9 (25.7)	39 (55.7)	31 (44.3)
Sibling	92 (18.6)	402 (81.4)	25 (44.6)	31 (55.4)	45 (50.0)	45 (50.0)	64 (27.8)	166 (72.2)
Other relative	144 (29.9)	338 (70.1)	27 (56.3)	21 (43.8)	47 (61.8)	29 (38.2)	75 (33.6)	148 (66.4)
Friend	40 (6.3)	591 (93.7)	24 (28.2)	61 (71.8)	44 (34.1)	85 (65.9)	40 (13.0)	268 (87.0)
Neighbor	166 (50.9)	160 (49.1)	18 (52.9)	16 (47.1)	34 (61.8)	21 (38.2)	56 (35.4)	102 (64.6)
Colleague	90 (17.7)	419 (82.3)	15 (13.9)	93 (86.1)	40 (32.3)	84 (67.7)	54 (24.2)	169 (75.8)
Person in the same situation	93 (57.4)	69 (42.6)	18 (56.3)	14 (43.8)	26 (61.9)	16 (38.1)	37 (45.7)	44 (54.3)
Professional of an institution	120 (60.3)	79 (39.7)	19 (36.5)	33 (63.5)	28 (56.0)	22 (44.0)	50 (58.1)	36 (41.9)
Professional of social services	125 (70.2)	53 (29.8)	16 (23.9)	51 (76.1)	23 (60.5)	15 (39.5)	47 (61.8)	29 (38.2)

NOTE: N=number of participants, %=percentage, Y= year, M=month, W= week, AEd=almost everyday
